# A designathon to co-create a sleep health communication package in adolescents

**DOI:** 10.1192/j.eurpsy.2025.2340

**Published:** 2025-08-26

**Authors:** H. F. Sit, A. H. Pang, W. Y. Chan, F. T. W. Cheung, S. X. Li

**Affiliations:** 1 Department of Psychology; 2 School of Public Health, The University of Hong Kong; 3 Department of Psychology, The Education University of Hong Kong; 4 The State Key Laboratory of Brain and Cognitive Sciences, The University of Hong Kong, Hong Kong, Hong Kong

## Abstract

**Introduction:**

Sleep is essential for one’s physical, emotional, and social well-being. Healthy sleep is particularly important for adolescents, individuals who undergo drastic developmental changes, making them susceptible to psychiatric disorders. Education and sleep health promotion to the public are urgently needed to improve population sleep health (Lim *et al.* Lancet Public Health 2023; 8 e820-e826). One novel way to improve sleep health communication is to engage adolescents with lived experiences of sleep disturbances in the development of sleep health innovations.

**Objectives:**

The designathon is a participatory innovation context that brings individuals to co-create communication packages to address a specific challenge within a short period. We aimed to present the process of crowdsourcing designathon to create sleep health communication packages for adolescents and the products produced in the event.

**Methods:**

The designathon was a two-day event held at a university building in Hong Kong. Adolescents were recruited from a summer programme about sleep and psychology and sorted into groups with a balance of educational backgrounds. A judging panel that included experts in sleep and mental health and a youth representative evaluated the communication packages. The products were scored based on content, accuracy, visuals, innovation, and delivery.

**Results:**

A total of 13 participants participated in the designathon. About 61.5% (n=8) of participants reported poor sleep quality. All three groups successfully created a sleep health communication package consisting of a poster, a leaflet, and a slideshow during the 2-day designathon event. One finalist was selected. The finalist package was a comprehensive package comprising psychoeducation and action elements to promote napping in school to address insufficient sleep and psychological health, and was described as structured, interesting, and informative although some information may be too technical to layperson based on judges’ comments.

**Conclusions:**

Designathon is a novel and successful strategy to engage the community to co-create sleep health communication package for adolescents. It is promising to utilise crowdsourcing designathon to increase access to sleep educational information and improve knowledge in the public. Future studies are needed to evaluate the feasibility, impact, and implementation of the products.

**Disclosure of Interest:**

None Declared

